# Supplementary material for: The Evolution and Characterization of the RNA Interference Pathways in Lophotrochozoa
Source: Genome Biol Evol. 2024 May 7;16(5):evae098. doi: 10.1093/gbe/evae098 (PMC11114477; doi:10.1093/gbe/evae098)
Supplement: evae098_Supplementary_Data [file evae098_supplementary_data.zip › Supplementary_materials.zip!/selected_miRNAs/NoGe_selected_miRNAs/mirdeep_pdf_files/NMRB01000380.1_Notospermus_geniculatus_strain_Ushimado_scaffold380,_whole_genome_shotgun_sequence_11915.pdf]

Provisional ID	:	NMRB01000380.1_Notospermus_geniculatus_strain_Ushimado_scaffold380,_whole_genome_shotgun_sequence_11915
Score total	:	2359.8
Score for star read(s)	:	3.9
Score for read counts	:	2350.9
Score for mfe	:	2
Score for randfold	:	0
Score for cons. seed	:	3
Total read count	:	4623
Mature read count	:	4485
Loop read count	:	0
Star read count	:	138

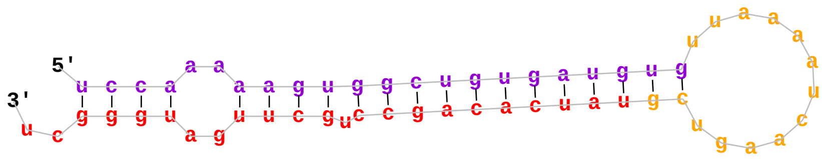

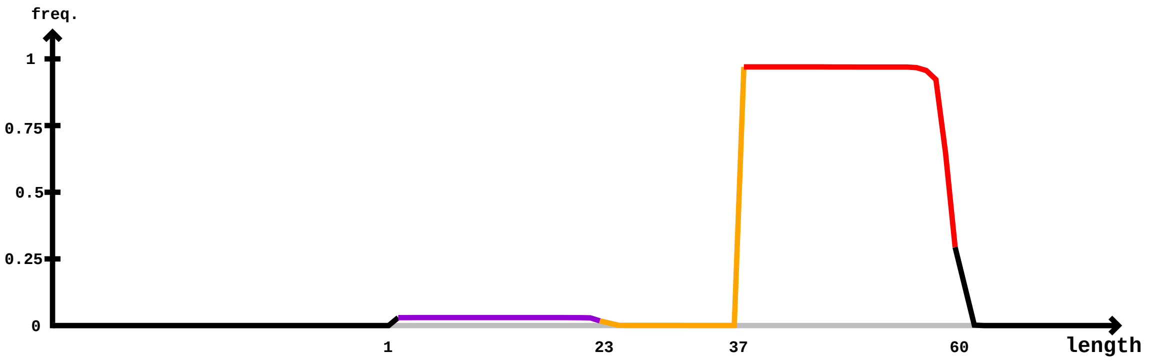

Star Mature

```
5'- ugguguacuagccgugcauuguauugcuucguauuccaaaaaguggcugugauguguuaaaaucaagucguaucacagccugcuugaugggcuauguggcaauacucuu   -3'   obs
    ugguguacuagccgugcauuguauugcuucguauuccaaaaaguggcugugauguguuaaaaucaagucguaucacagccugcuugaugggcuauguggcaauacucuu         exp
    .(((((((.....)))))))((((((((.((((.((((..((((((((((((((((............)))))))))))).))))..)))).)))).))))))))....    reads   mm   sample
    ..................................uccaaaaaguggcuguga.........................................................    1       0    Mm3
    ..................................uccaaaaaguggcugugaug.......................................................    1       0    Mm3
    ..................................uccaaaaaguggcugugaugu......................................................    25      0    Mm3
    ..................................uccaaaaaguggcugugaugA......................................................    1       1    Mm3
    ..................................uccaaaaaguggcugugauAug.....................................................    1       1    Mm3
    ..................................uccaaaaaguggcuguCaugug.....................................................    1       1    Mm3
    ..................................uccaaaaaguggcugugaugug.....................................................    13      0    Mm3
    ..................................uccaaaaaguggcugugauguU.....................................................    1       1    Mm3
    ..................................uccaaaaaguggcuguAaugugu....................................................    1       1    Mm3
    ..................................uccaaaaaguggcugugaugugu....................................................    2       0    Mm3
    ..................................uccaaaaaguggcugugaugugG....................................................    1       1    Mm3
    ....................................caaaaaguggcugugaugu......................................................    1       0    Mm3
    ........................................................uuaaaaucaagucguaucacagc..............................    1       0    Mm3
    ......................................................................uaucacagccugcuugau.....................    1       0    Mm3
    ......................................................................uaucacagccugcuugaug....................    1       0    Mm3
    ......................................................................uaucacagccugcuugaugU...................    1       1    Mm3
    ......................................................................uaucacagccugcuugaGgg...................    1       1    Mm3
    ......................................................................uaucacagccAgcuugaugg...................    1       1    Mm3
    ......................................................................uaucacagccugcuugaugg...................    12      0    Mm3
    ......................................................................uaucacagccugcuugaAggg..................    1       1    Mm3
    ......................................................................uaucacagccCgcuugauggg..................    1       1    Mm3
    ......................................................................uaucacagccugcuugauggg..................    116     0    Mm3
    ......................................................................uaucacagccuUcuugauggg..................    1       1    Mm3
    ......................................................................uaucacagccugUuugauggg..................    1       1    Mm3
    ......................................................................uaucacagccugcuugCuggg..................    1       1    Mm3
    ......................................................................uaucacagccugcuugauggC..................    1       1    Mm3
    ......................................................................uaucacagUcugcuugauggg..................    1       1    Mm3
    ......................................................................uaucacagccugcuugaugggA.................    10      1    Mm3
    ......................................................................uaucacGgccugcuugaugggc.................    1       1    Mm3
    ......................................................................uaucacagccCgcuugaugggc.................    1       1    Mm3
    ......................................................................uaucacagccugcuugaugggG.................    1       1    Mm3
    ......................................................................uaucacagccugcuugaugAgc.................    2       1    Mm3
    ......................................................................uaucacagccugcuugGugggc.................    1       1    Mm3
```

```
                                  Star                                  Mature
ugguguacuagccgugcauuguauugcuucguauuccaaaaaguggcugugauguguuaaaaucaagucguaucacagccugcuugaugggcuauguggcaauacucuu
......................................................................uaucacagUcugcuugaugggc.................   1     1   Mm3
......................................................................uaAcacagccugcuugaugggc.................   1     1   Mm3
......................................................................uaucacagccugcuugaAgggc.................   1     1   Mm3
......................................................................uaucacaUccugcuugaugggc.................   1     1   Mm3
......................................................................uaucacagccugcuugaugggc.................   147   0   Mm3
......................................................................uaucacagccugcuugaCgggc.................   1     1   Mm3
......................................................................uaucacagccugcuugaugggU.................   2     1   Mm3
......................................................................uaucacagccugcuugaGgggcu................   1     1   Mm3
......................................................................uaucacagcUugcuugaugggcu................   2     1   Mm3
......................................................................uaucacagccugcuugaugggcu................   117   0   Mm3
......................................................................uaucacagUcugcuugaugggcu................   2     1   Mm3
......................................................................uaucacagccugcuugaugggcG................   6     1   Mm3
......................................................................uaucacagccugcuugaugggcC................   1     1   Mm3
......................................................................uaucacagccugcuugaugggAu................   1     1   Mm3
......................................................................uaucacagccugcuugaugggcuU...............   1     1   Mm3

..................................uccaaaaaguggcugugaug.......................................................   1     0   Fm3
..................................uccaaaaaguggAugugaug.......................................................   1     1   Fm3
..................................uccaaaaaguggcugugaugu......................................................   9     0   Fm3
..................................uccaaaaaguggcugugaugug.....................................................   7     0   Fm3
..................................uccaaaaaguggcugugauguU.....................................................   1     1   Fm3
..................................uccaaaaaguggcugugaugugu....................................................   4     0   Fm3
..................................uccaaaaaguggcugugauguguu...................................................   2     0   Fm3
......................................................................uaucacagccugcuugau.....................   4     0   Fm3
......................................................................uaucacagccAgcuugaug....................   3     1   Fm3
......................................................................uaucacagccugcuugaug....................   33    0   Fm3
......................................................................uaucacagccugcuugaugg...................   87    0   Fm3
......................................................................uaucacagccugcuugaCgg...................   1     1   Fm3
......................................................................uaucacagccugcuugaugU...................   3     1   Fm3
......................................................................Caucacagccugcuugauggg..................   1     1   Fm3
......................................................................uaucacagccugcuugUuggg..................   2     1   Fm3
......................................................................uaucacagccugcuugCuggg..................   1     1   Fm3
......................................................................uUucacagccugcuugauggg..................   1     1   Fm3
......................................................................uaucCcagccugcuugauggg..................   1     1   Fm3
......................................................................uaucacagccCgcuugauggg..................   1     1   Fm3
......................................................................uaucacagccugcuugaCggg..................   5     1   Fm3
......................................................................uaucacagcAugcuugauggg..................   1     1   Fm3
......................................................................uaucacagccugcuugaAggg..................   6     1   Fm3
......................................................................uaucacagccGgcuugauggg..................   1     1   Fm3
......................................................................uaAcacagccugcuugauggg..................   1     1   Fm3
......................................................................uGucacagccugcuugauggg..................   2     1   Fm3
......................................................................uaucacagcUugcuugauggg..................   9     1   Fm3
......................................................................uaucacagccugcuugauggg..................   644   0   Fm3
......................................................................uaucacagccugcuugaGggg..................   6     1   Fm3
......................................................................uaucacagccugcuugauUgg..................   2     1   Fm3
......................................................................uaucacagccugcuugaAgggc.................   1     1   Fm3
......................................................................uaucacagccugcuugaGgggc.................   5     1   Fm3
......................................................................uaucacagccugcuugCugggc.................   1     1   Fm3
......................................................................uaucacagcUugcuugaugggc.................   4     1   Fm3
......................................................................uaucacaUccugcuugaugggc.................   1     1   Fm3
......................................................................uaucacagccugcuugaugUgc.................   1     1   Fm3
......................................................................uaucacagccugcuugauUggc.................   1     1   Fm3
......................................................................uaucGcagccugcuugaugggc.................   1     1   Fm3
......................................................................uaucacaAccugcuugaugggc.................   2     1   Fm3
......................................................................uaucacagccugcuugUugggc.................   1     1   Fm3
......................................................................uaucacagccugcuugaugAgc.................   10    1   Fm3
......................................................................uaucacagccugcuugGugggc.................   1     1   Fm3
......................................................................uaucacagccugcuugauCggc.................   1     1   Fm3
......................................................................uaucacagccugcuugaCgggc.................   4     1   Fm3
......................................................................uaucacagccugcuugaugggA.................   96    1   Fm3
......................................................................Naucacagccugcuugaugggc.................   1     1   Fm3
......................................................................uaucacagccAgcuugaugggc.................   3     1   Fm3
......................................................................uauAacagccugcuugaugggc.................   1     1   Fm3
......................................................................uaucacagccuAcuugaugggc.................   1     1   Fm3
......................................................................uaucacagccugcuugaugggU.................   9     1   Fm3
......................................................................uaucacagccugcuugaugggc.................   614   0   Fm3
......................................................................uaucacagccugcuugaugggcA................   9     1   Fm3
......................................................................uaucacagcUugcuugaugggcu................   7     1   Fm3
......................................................................uaucacagccugcuugaGgggcu................   2     1   Fm3
......................................................................uaucacagccugcuugaCgggcu................   6     1   Fm3
```

Star **Mature**

```
ugguguacuagccgugcauuguauugcuucguauuccaaaaaguggcugugauguguuaaaaucaagucguaucacagccugcuugaugggcuauguggcaauacucuu
......................................................................uaucacagccugcuugaAgggcu................   6     1   Fm3
......................................................................uaucacagccugcuugaugggcu................   675   0   Fm3
......................................................................uaucacagccGgcuugaugggcu................   1     1   Fm3
......................................................................uaucacagccugcuuAaugggcu................   1     1   Fm3
......................................................................uaucacagccugcuugCugggcu................   2     1   Fm3
......................................................................uaucacagccugcuugaugggAu................   19    1   Fm3
......................................................................uaucacagccugcuugaugAgcu................   1     1   Fm3
......................................................................uaucaUagccugcuugaugggcu................   1     1   Fm3
......................................................................uauUacagccugcuugaugggcu................   1     1   Fm3
......................................................................uaucacagccugcuugaugggcuU...............   8     1   Fm3
......................................................................uaucacagccugcuugaugggcua...............   4     0   Fm3
......................................................................uaucacagccugcuugaugggcuUu..............   2     1   Fm3

..................................uccaaaaaguggcugugaugu......................................................   1     0   Mm1
..................................uccaaaaaguggcugugaugug.....................................................   3     0   Mm1
......................................................................uaucacagccugcuugaugg...................   12    0   Mm1
......................................................................uaucacagccugcuugauggg..................   52    0   Mm1
......................................................................uaucacagccugcuugaugAgc.................   2     1   Mm1
......................................................................uaucacagccugcuugaCgggc.................   1     1   Mm1
......................................................................uaucacagccAgcuugaugggc.................   1     1   Mm1
......................................................................uaucacagccugcuugaugggc.................   69    0   Mm1
......................................................................uaucacagccugcuugaAgggc.................   1     1   Mm1
......................................................................uaucacagccugcuugaGgggcu................   1     1   Mm1
......................................................................uaucacagccugcuugaugggcG................   1     1   Mm1
......................................................................uaucacagccugcuugCugggcu................   1     1   Mm1
......................................................................uaucacagccugcuugaAgggcu................   1     1   Mm1
......................................................................uaucacagccugcuugaugggcA................   5     1   Mm1
......................................................................uaucacagccugcuugaugAgcu................   4     1   Mm1
......................................................................uaucacagccugcuugaugUgcu................   1     1   Mm1
......................................................................uaucacagccugcuugaugggcu................   87    0   Mm1

..................................uccaaaaaguggcugugau........................................................   1     0   Mm2
..................................uccaaaaaguggcugugaugu......................................................   2     0   Mm2
..................................uccaaaaaguggcugugauguU.....................................................   1     1   Mm2
..................................uccaaaaaguggcugugaugug.....................................................   11    0   Mm2
..................................uccaaaaaguggcugugaugugu....................................................   3     0   Mm2
..................................uccaaaaaguggcugugauguguuaaaauc.............................................   1     0   Mm2
..........................................................aaaaucaagucguaucacagccug...........................   1     0   Mm2
......................................................................uaucacagccugcuugau.....................   1     0   Mm2
......................................................................uaucacagccugcuugaug....................   7     0   Mm2
......................................................................uaucacagccugcuugauU....................   1     1   Mm2
......................................................................uaucacagccugcuugaGg....................   1     1   Mm2
......................................................................uaucacagccugcuugaugg...................   11    0   Mm2
......................................................................uaucacagccugcuugaugU...................   2     1   Mm2
......................................................................uaucacagccAgcuugauggg..................   1     1   Mm2
......................................................................uaucacagccugcuugUuggg..................   1     1   Mm2
......................................................................uaucacagccugcuugauggC..................   2     1   Mm2
......................................................................uaucacagccugcuugaCggg..................   1     1   Mm2
......................................................................uaucacagccugcuugauggg..................   203   0   Mm2
......................................................................uaucacagccugcuugaAggg..................   1     1   Mm2
......................................................................uaucacagccugcuugaugUg..................   1     1   Mm2
......................................................................uaucacagccugUuugauggg..................   1     1   Mm2
......................................................................uaucacagccugcuugaGggg..................   2     1   Mm2
......................................................................uaucacagccugcuugaugAg..................   1     1   Mm2
......................................................................uaucacagccugcuugUugggc.................   1     1   Mm2
......................................................................uaucacagccugcuugaugAgc.................   6     1   Mm2
......................................................................uGucacagccugcuugaugggc.................   1     1   Mm2
......................................................................uaucacagccugcuCgaugggc.................   1     1   Mm2
......................................................................uaucacagccugcuugaugggA.................   21    1   Mm2
......................................................................uaucacagccugcuugaugggG.................   2     1   Mm2
......................................................................uaucacagccugcuugaugggU.................   4     1   Mm2
......................................................................uaucacagccugcuugauCggc.................   1     1   Mm2
......................................................................uaucacagccugcuugaAgggc.................   1     1   Mm2
......................................................................uaucacagccugcuugaugggc.................   353   0   Mm2
......................................................................uaucacagccugcuugCugggc.................   1     1   Mm2
......................................................................uaucacagccugcuugaCgggcu................   1     1   Mm2
......................................................................uaucacagccugcuugaGgggcu................   1     1   Mm2
......................................................................uaucacagUcugcuugaugggcu................   1     1   Mm2
......................................................................uaucacagccugcuugaugggAu................   4     1   Mm2
......................................................................uaucacagccugcuugGugggcu................   1     1   Mm2
```

```
                                  Star                                  Mature
ugguguacuagccgugcauuguauugcuucguauuccaaaaaguggcugugaugugguaaaaaucaagucguaucacagccugcuugaugggcuauguggcaauacucuu
.......................................................................uaucacagccugcuugaugggcu................  236  0  Mm2
.......................................................................uUucacagccugcuugaugggcu................  1    1  Mm2
.......................................................................uaucacagccugcuugaugggcA................  2    1  Mm2
.......................................................................uaucacagccugcuugaugggcG................  7    1  Mm2
.......................................................................uaGcacagccugcuugaugggcu................  1    1  Mm2
.......................................................................uaucacagccugcuugaAgggcu................  3    1  Mm2
.......................................................................uaucacagccugcuugaugggcuU...............  10   1  Mm2
.......................................................................uaucacagccugcuugaugggcua...............  1    0  Mm2
.......................................................................uaucacagccugcuugaugggcuUu..............  2    1  Mm2

..................................uccaaaaaguggcugugUugu.......................................................  1    1  Fm1
.......................................................................uaucacagccugcuugaug....................  3    0  Fm1
.......................................................................uaucacagccugcuugaugg...................  6    0  Fm1
.......................................................................uaucacagccugcuugauggg..................  95   0  Fm1
.......................................................................uaucacagccugcuugaGgggc.................  1    1  Fm1
.......................................................................uaucacagccugcuugGugggc.................  1    1  Fm1
.......................................................................uaucacagccugcuugCugggc.................  1    1  Fm1
.......................................................................uaucacagccugcuugaAgggc.................  1    1  Fm1
.......................................................................uaucacagccugcuugaugAgc.................  7    1  Fm1
.......................................................................uaucacagccugcuugaugggU.................  3    1  Fm1
.......................................................................uauGacagccugcuugaugggc.................  1    1  Fm1
.......................................................................Naucacagccugcuugaugggc.................  1    1  Fm1
.......................................................................uaucacagccugcuugaugggc.................  171  0  Fm1
.......................................................................uaucacagccuCcuugaugggc.................  1    1  Fm1
.......................................................................uaucacagccugcuugaugggA.................  12   1  Fm1
.......................................................................uaucacagccugcuugaugggcA................  1    1  Fm1
.......................................................................uaucacagccugcuugaugggcu................  90   0  Fm1
.......................................................................uaucacagccugcuugaugggcG................  7    1  Fm1
.......................................................................uaucacagccugcuugaCgggcu................  1    1  Fm1
.......................................................................uaucacagccugcuugaugggAu................  1    1  Fm1
.......................................................................uaucacagccugcuugaugggcuU...............  6    1  Fm1
.......................................................................uaucacagccugcuugaugggcuUu..............  4    1  Fm1

..................................uccaaaaaguggcugugaugu.......................................................  11   0  Fm2
..................................uccaaaaaguAgcugugaugu.......................................................  1    1  Fm2
..................................uccaaaaaguggcugugaugug......................................................  29   0  Fm2
.......................................................................uaucacagccugcuuUau.....................  5    1  Fm2
.......................................................................uaucacagccugcuuAaugg...................  1    1  Fm2
.......................................................................uaucacagccugcuugaugg...................  20   0  Fm2
.......................................................................uaucacagccugcuugaAggg..................  1    1  Fm2
.......................................................................uaucacagccugcuugaCggg..................  1    1  Fm2
.......................................................................uaucacagccugcuugUuggg..................  1    1  Fm2
.......................................................................uaucaAagccugcuugauggg..................  1    1  Fm2
.......................................................................uaucacagccugcuugauggg..................  96   0  Fm2
.......................................................................uaucacagccugcuugauggU..................  1    1  Fm2
.......................................................................uaucacagccugcuugaugggc.................  20   0  Fm2
.......................................................................uaucacagccugcuugaugggA.................  18   1  Fm2
.......................................................................uaucacagccugcuugaugAgc.................  6    1  Fm2
.......................................................................uaucacagccugcuugaugCgcu................  1    1  Fm2
```
